# The interplay between perceived fatigability, intrinsic capacity, and physical activity: network analysis in a British birth cohort study

**DOI:** 10.1093/gerona/glaf192

**Published:** 2025-09-02

**Authors:** Kaisa Koivunen, Lotta Palmberg, Gabriela Lunansky, Almar Kok, Nancy W Glynn, Rachel Cooper

**Affiliations:** Faculty of Sport and Health Sciences and Gerontology Research Center, University of Jyväskylä, Jyväskylä, Finland; Faculty of Sport and Health Sciences and Gerontology Research Center, University of Jyväskylä, Jyväskylä, Finland; Department of Public Health, University of Turku and Turku University Hospital, Turku, Finland; Centre for Population Health Research, University of Turku and Turku University Hospital, Turku, Finland; Department of Epidemiology and Data Science, Amsterdam UMC, Vrije Universiteit Amsterdam, Amsterdam, The Netherlands; Aging & Later Life program, Amsterdam Public Health, Amsterdam, The Netherlands; Department of Epidemiology and Data Science, Amsterdam UMC, Vrije Universiteit Amsterdam, Amsterdam, The Netherlands; Aging & Later Life program, Amsterdam Public Health, Amsterdam, The Netherlands; Department of Epidemiology, Center for Aging and Population Health, School of Public Health, University of Pittsburgh, Pittsburgh, Pennsylvania, United States; AGE Research Group, Translational and Clinical Research Institute, Faculty of Medical Sciences, Newcastle University, Newcastle upon Tyne, United Kingdom; NIHR Newcastle Biomedical Research Centre, Newcastle upon Tyne Hospitals NHS Foundation Trust, Cumbria, Northumberland, Tyne and Wear NHS Foundation Trust, and Faculty of Medical Sciences, Newcastle University, Newcastle upon Tyne, United Kingdom

**Keywords:** Fatigue, Disablement process, Functional capacity, Complex systems

## Abstract

**Background:**

Fatigability—an individual’s susceptibility to fatigue when performing standardized activities—may arise from diminished functional reserves and contribute to reduced physical activity (PA) levels, potentially reinforcing the disablement process. In this study, we used network analysis to examine the associations among physical and mental fatigability, different domains of intrinsic capacity (IC), and PA. Additionally, we investigated whether fatigability mediates the association between IC and PA.

**Methods:**

We ran analyses of cross-sectional data on participants from the MRC National Survey of Health and Development at age 68-69 years (*n* = 1537). Physical and mental fatigability were assessed using the Pittsburgh Fatigability Scale, and PA was self-reported. We quantified 5 IC domains: vitality, locomotion, cognition, psychology, and sensory, using performance-based and self-reported measurements. Networks were estimated based on mixed graphical models stratified by sex.

**Results:**

In both sexes, greater physical and mental fatigability were consistently associated with lower scores in 2 IC domains (psychology and locomotion) and PA. The network structure showed that physical fatigability mediated the relation between locomotion and psychology domains and PA. The same applied to mental fatigability, but to a lesser extent and only in women.

**Conclusions:**

Perceived physical fatigability is a potentially important factor on the pathway between lower physical and mental resources and activity behavior in older adults. Future work is needed to study the temporality of these associations.

## Introduction

Fatigability, defined as the level of fatigue experienced during standardized activities,^1^ may play a central role in age-related functional decline, potentially contributing to disability and death.^2^ Reduced physiological reserves, such as neuromuscular strength, cardiovascular endurance, and metabolic efficiency, limit the energy available for daily activities, resulting in increased physical and/or mental fatigue during tasks.^3^ This often leads to behavioral adaptations aimed at conserving energy, including reduced physical activity levels, which may further accelerate functional decline. In support of this theory, a survey of 754 nondisabled community-dwelling adults aged 70 years and older found that the main reason for restricting activity was fatigue.^4^ In addition, Qiao et al.^5^ found that greater perceived physical fatigability explained the association between lower physical activity and slower gait speed in older adults. These findings suggest that fatigability may be a key mechanism linking declining functional capacity with reduced physical activity in later life.

Functional capacity, referring to an individual’s maximum potential to perform under ideal conditions, is a broad concept and is operationalized in myriad ways across studies. Consequently, the associations between fatigability, physical activity, and functional capacity remain unclear. One way to address this is to adopt the concept of intrinsic capacity (IC) in conjunction with network analysis to clarify the multitude of associations between these factors. IC is defined by the World Health Organization (WHO) as “the composite of all the physical and mental capacities that an individual can draw on at any point in time” and encompasses 5 key domains: locomotion, cognition, psychology, sensory function, and vitality. These domains typically decline with age and are linked to adverse health outcomes. Together with the environment, they determine functional ability (ie, the actual performance of tasks and activities in daily life) and shape the trajectory of healthy aging.^6^ Although physical fatigability has been linked to physical activity and mobility (ie, locomotion),^5^^,^^7^^,^^8^ its associations with other domains of IC, and with mental fatigability, remain less well understood, although these factors may be interlinked. For instance, Palmberg et al.^9^ found that individuals with more fragmented physical activity patterns report higher levels of both physical and mental fatigability. This suggests that both can lead to maladaptive physical activity behavior, with links to mental fatigability possibly acting via perceived effort or cognitive resources.^10^ Therefore, gaining a better understanding of these interrelations, many of which may be bi-directional, would provide more comprehensive insights into fatigability and its potential mediating role between IC and physical activity. This may help to identify potentially meaningful targets for intervention to promote healthy aging.

Intrinsic capacity is typically studied by summing the measures within each domain and then combining the scores for each of the 5 domains into one overall score, which does not capture the interplay between different body functions.^11^ Recently, we demonstrated that IC can be conceptualized and analyzed as a complex network system.^12^ Building on the disablement process framework, we extended this approach and used network analysis in a British birth cohort to investigate the associations among the 5 domains of IC and physical activity, as well as to examine whether fatigability mediates these associations. Network analysis, a graphical statistical technique, offers multiple advantages for studying complex, interconnected phenomena.^13^ For example, it enables the simultaneous examination of direct and indirect associations among IC domains, fatigability, and physical activity as a connected system, rather than isolated pairwise relationships. One key feature of network analysis is the use of centrality measures, which we used to explore how strongly fatigability is connected to other nodes, providing insights into its role within the system.

## Methods

### Study participants

Data were drawn from the Medical Research Council National Survey of Health and Development (NSHD).^14–16^ The NSHD cohort originally consisted of 5362 male and female singleton births within marriage, born within 1 week in March 1946 in mainland Britain. NSHD participants have been followed up across life. In this study, we used data collected in the 24th assessment in 2014 and 2015, when the target sample was 2816 study members still living in mainland Britain. Participants who had died prior to the assessment were excluded from the sample, and individuals who had emigrated, were lost to follow-up and remained untraced, or had previously requested not to be contacted were not invited. A total of 2148 participants completed a postal questionnaire at age 68 years and received a nurse home visit at age 69, representing 76% of the remaining target population.^15^ Ethical approval for the 2014-2015 assessment was provided by the Queen Square Research Ethics Committee (14/LO/1073) and the Scotland A Research Ethics Committee (14/SS/1009). Study participants provided written informed consent.

### Physical and mental fatigability

Physical and mental fatigability were assessed in the postal questionnaire using the Pittsburgh Fatigability Scale (PFS).^17^ The PFS and the selected cutoffs applied for higher (ie, more severe) fatigability have been validated in US populations with similar characteristics, in terms of age and sex distribution to the NSHD and, so the demonstrated construct and predictive validity, and reliability are likely to apply in UK populations as well.^8^^,^^10^^,^^17^ In this assessment, the participants were asked to rate the level of physical and mental fatigue they would expect or imagine they would feel after completing 10 activities ranging from activities expected to be low intensity (eg, “watching TV for 2 hours”) to those viewed as high intensity (eg, “moderate-to-high-intensity strength training for 30 minutes”). The response options ranged from 0 (no fatigue) to 5 (extreme fatigue). The responses for each item were summed to obtain a separate total subscale score for physical and mental fatigability, each ranging from 0 to 50 (higher = greater fatigability). Additionally, a cut point of 15 or greater on the PFS Physical score was used to denote more severe perceived physical fatigability, whereas a cut point of 13 or greater on the PFS Mental score indicates more severe perceived mental fatigability.^8^^,^^17^

If ≤3 items were missing, but a related question on whether the activity had been performed in the past month was available, the values of the missing responses were imputed from the mean of the individual’s valid responses and adjusted to account for variation in intensity levels of different activities and differences in fatigue levels of those who had or had not performed each activity. This rule-based approach applies correction factors derived from observed patterns in the data and the method has previously been shown to maintain the distribution of PFS scores (see the Supplement in Cooper et al., 2019 for details). We performed the imputation for 314 (20%) participants for physical fatigability and for 386 (25%) participants for mental fatigability.

### Intrinsic capacity

Intrinsic capacity was operationalized using variables aligned with the 5 key domains defined by the WHO. Additionally, the criteria for selecting indicators included guidance from working group publications (if available), relevance to factors important for functional decline, the ability to capture the full continuum of capacity when possible, and the necessity to maintain statistical power by limiting the number of variables to 1 or 2 per domain.

*Vitality* is defined as “a physiological state resulting from the interaction between multiple physiological systems, reflected in (the level of) energy and metabolism, neuromuscular function, and immune and stress response functions of the body.”^18^ We quantified vitality using grip strength, which is a validated and widely used indicator of neuromuscular function, and is associated with functional limitations.^19^ Grip strength was measured twice in each hand using a Jamar digital dynamometer. The maximum value expressed in kilograms (kg) was used in analyses. Similarly to chair rise and gait speeds, the mean of the lowest sex-specific fifth was assigned to participants who were unable to perform the test for health reasons (*n* = 27).

*Locomotion* is described as “a state of the musculoskeletal system that encompasses endurance, balance, muscle strength, muscle function, muscle power and a joint function of the body.”^20^ Therefore, locomotion represents the expression of physiological capacity, involving complex movements that require not only strength but also coordination, balance, and endurance. The locomotion domain was measured using chair rise and gait speed tests. In the chair rise test, participants were instructed to rise from a chair and sit back down 10 times as fast as possible, or 5 times if the participant could not complete 10. The chair rise speed (stands/min) was calculated based on the number of rises completed and the time recorded. Walking time at one’s usual pace was measured over 2.44 m (8 ft), from which gait speed (m/s) was calculated. In line with the previous studies in this cohort,^21^^,^^22^ to maintain the representativeness of the study sample, we assigned the mean of the lowest sex-specific fifth for chair rise speed and gait speed to participants who were unable to perform the test for health reasons (chair rise speed *n* = 76 and gait speed *n* = 42).

*Cognitive domain* encompasses a wide range of cognitive functions, including memory, attention, processing speed, executive function, and perception.^23^ In this study, we operationalized the cognitive domain using verbal memory and information processing speed, which are sensitive to cognitive aging^24^ and associated with adverse health outcomes.^25^ Verbal memory was assessed using a 15-item word list. Each word was presented for 2 seconds before participants were instructed to write down as many words as they could remember. This was repeated over 3 identical trials, and the number of words recalled during each trial was summed (maximum 45). Information processing speed was assessed with a visual search speed task, in which participants were instructed to identify and cross out all letters P and W randomly placed in a grid of other letters within 1 minute. The score is the total number of letters searched (maximum 600).

*The psychological domain* emphasizes internal resources that enable individuals to adapt to age-related changes. It focuses not only on mitigating declines but also on enhancing emotional well-being and perceived control.^26^ This was indicated by the Pearlin Mastery Scale,^27^ which measures the extent to which a person perceives being in control of one’s life events and ongoing situations. The scale comprises 5 negatively worded items and 2 positively worded items with the following Likert response options ranging from 1 (strongly disagree) to 4 (strongly agree). The negatively worded items were reverse-coded before scoring, resulting in a score ranging from 7 to 28, with higher scores indicating greater levels of mastery.

*The sensory domain* includes vision and hearing, which are essential for maintaining functional independence.^6^ Vision and hearing were measured using 2 specific questions, which are valid and practical indicators for older age groups and are often associated with adverse outcomes.^28^ Near vision was assessed with a question: “In the last 12 months, have you had difficulty with reading a newspaper?” and hearing “In the last 12 months, have you had difficulty with hearing conversation in a noisy room?” The response options to both questions were 1 (no difficulty), 2 (a little difficulty), 3 (some difficulty), and 4 (a great deal of difficulty). For statistical analyses, the responses were categorized as 1 (no difficulty) to 3 (some/a great deal of difficulty) to ensure adequate categories while maintaining meaningful distinctions between individuals with and without functional sensory limitations.

### Physical activity

Participants were asked to report whether they had taken part in any sports, vigorous leisure activities, or exercises in their leisure time, not including getting to and from work, in the last 4 weeks and, if so, on how many occasions. As in previous work,^29^ the responses were categorized as follows: none (reported no participation), participation 1-4 times in the past 4 weeks, and participation ≥5 times in the past 4 weeks.

### Covariates

We included sex, socioeconomic position (SEP; measured by educational attainment), cigarette smoking, BMI, and long-term health conditions as covariates in the analyses. As all participants were the same age, age was not included. As formal education is normally completed in young adulthood, we used the highest educational level achieved by age 26 years as a proxy for SEP. Education was coded into 4 categories: (1) no qualifications, (2) up to O-level or equivalent (usually attained at age 16), (3) A-level or equivalent (usually attained at age 18), and (4) degree or higher. Information on cigarette smoking was obtained by asking participants whether they currently smoked cigarettes, and if the response was negative, whether they had ever smoked cigarettes. The answers were used to construct a smoking status variable with 3 categories coded as (1) current, (2) former, and (3) never smoker. BMI (kg/m^2^) was calculated from height (m) and weight (kg) measured by a trained nurse during the clinical assessment at age 69. Number of long-term health conditions were ascertained based on responses to questions on doctor-diagnoses of 19 long-term health conditions, which were classified for the analyses as (1) no long-term health conditions, (2) 1-2, and (3) 3 or more.

### Statistical analysis

Descriptive analyses were conducted with IBM SPSS statistics 28. Spearman correlations between the study variables and the network estimations were performed using the statistical programming language R (4.3.0) and its available packages. All analyses were performed stratified by sex due to differences between men and women in most of the main variables, especially grip strength levels. Because physical and mental fatigability scores were strongly correlated (*r*s = 0.71 for both men and women, Figures S1 and S2, see online supplementary material for a color version of this figure), we ran the network analyses separately for physical and mental fatigability to isolate the unique associations between each type of fatigability and other variables. The utilized network estimation needs full information, and the final analytical sample consisted of 1537 participants in the networks including physical fatigability and 1440 participants in the networks including mental fatigability.

#### Network analysis

Network analysis provides a data-based and graphical approach to investigate the simultaneous relationships between the proposed variables of a system. In the network graphs, nodes represent the variables and edges (lines) the statistical association between 2 variables (circles). In this study, we estimated networks based on Mixed Graphical Models (MGM) using the *mgm* package in R^30^ following the reporting standards for network analyses in cross-sectional data.^31^ The MGM networks are extensions of correlation and partial correlation models, which can accommodate binary, ordinal, and continuous variables. In our MGM networks, all associations represent pairwise interactions, that is, (*k* = 2) interactions that in the network analysis indicate conditional association. The associations between 2 nodes were estimated while controlling for all other nodes, meaning that the absence of a relation between 2 variables indicates that those 2 variables are conditionally independent given all other variables in the network. In this study, positive associations are represented with green edges, negative associations with red edges, and associations involving categorical variables with grey edges.

The “least absolute shrinkage and selection operator” (LASSO)^32^ was used to limit the estimation of false positive associations between nodes as it shrinks all edge weights toward zero and sets small weights to exactly zero. The LASSO utilizes an Extended Bayesian Information Criterion (EBICglasso) tuning parameter to control the degree to which regularization is performed. In the EBIC computation, a hypertuning parameter is used to penalize the model complexity, and it is typically set between 0 and 0.5 with higher values preferring simpler models (ie, more parsimonious models with fewer edges).^33^ In the reported models, the tuning parameter was set at 0.5 to ensure a more conservative network estimation. To ensure consistent visual comparison between the networks for men and women, we fixed the maximum edge weight to the largest edge weight observed across both networks. The *qgraph* R package^34^ was used to visualize the networks with the Fruchterman–Reingold algorithm, which sets the strongly associated nodes close to each other.^35^

We first estimated networks including only physical or mental fatigability, physical activity, and IC domains, and then similar networks adjusted for covariates (education, cigarette smoking, long-term health conditions, and BMI). Additionally, we calculated the predictability of each variable in the network by other variables directly connected to it. For numerical variables, the proportion of explained variance (*R*^2^), and for categorical variables, the proportion of correct classification (CC) were calculated. These measures were visualized with a pie chart around the respective node.^36^

To examine the accuracy of the edge weights, we conducted nonparametric bootstrapping analyses (ie, resampling data with replacement, 1000 iterations) using the “bootnet” function in the *bootnet* R package.^33^ The stability plots in the Supplement display the edge estimates in the study sample and the mean edge estimates in the bootstrapped samples with the 95% confidence interval band from the bootstrapped edge weights. Node centrality, indicating the importance of the nodes within a network, was computed with strength centrality (*the total weight of connections a node has with other nodes*).^33^ Nodes with more or stronger edges with neighboring nodes will be positioned more central and could play an important role within the network system.

To further test the potential mediating role of physical and mental fatigability between the IC variables and physical activity, as identified from the network models, we used the structural equation model (SEM) framework to conduct mediation analyses with the *lavaan* R^37^ package. Each association was tested in separate models, first unadjusted and then adjusted for all other variables in the network model.

## Results

The characteristics of the analytical sample (*n* = 1537) are reported in Table 1. Men had lower mean physical and mental fatigability scores than women and a lower prevalence of high perceived fatigability (PFS: 40% vs 60%; MFS: 37% vs 63%; both *P* < .001). Men also had faster gait speed and chair rise speed, higher grip strength, and greater mastery, whereas women performed better on verbal memory and visual search tasks. Most participants reported no near vision impairment, and over half reported no hearing difficulties, though reports of some or a great deal of hearing difficulty were slightly more common among men. Men were more likely to have higher education and a history of smoking. There were no sex differences in BMI, physical activity, or number of long-term health conditions.

**Table 1.** Characteristics of the NSHD participants at age 68-69 years included the analytic sample by sex.

	Men (*n* = 743)	Women (*n* = 794)	*P* value^a^
	Mean ± SD	Mean ± SD	
**PFS physical score, 0-50**	12.6 ± 8.4	15.9 ± 9.2	**<.001**
**PFS mental score, 0-50**	6.8 ± 7.1	9.4 ± 8.1	**<.001**
**Gait speed (m/s)**	1.13 ± 0.30	1.08 ± 0.28	**<.001**
**Chair rise speed (stands per minute)**	27.8 ± 8.6	26.3 ± 8.1	**<.001**
**Verbal memory**	21.7 ± 5.9	23.8 ± 5.8	**<.001**
**Visual search**	266.5 ± 70.1	273.5 ± 68.8	**.040**
**Mastery**	22.8 ± 3.3	21.6 ± 3.6	**<.001**
**Grip strength (kg)**	40.7 ± 8.5	24.6 ± 5.7	**<.001**
**BMI (kg/m^2^)**	27.9 ± 4.3	27.9 ± 5.5	.788
	***n* (%)**	***n* (%)**	
**PFS physical ≥ 15**	261 (40)	396 (60)	**<.001**
**PFS mental ≥ 13**	126 (37)	214 (63)	**<.001**
**Near vision**			**.002**
** No difficulty**	702 (95)	721 (91)	
** A little difficulty**	32 (4)	42 (5)	
** Some or a great deal of difficulty**	9 (1)	31 (4)	
**Hearing in conversation**			**.033**
** No difficulty**	374 (50)	446 (56)	
** A little difficulty**	212 (29)	216 (27)	
** Some or a great deal of difficulty**	157 (21)	132 (17)	
**Physical activity**			.144
** None**	394 (53)	427 (54)	
** 1-4 times/month**	99 (13)	129 (16)	
** 5 or more times/month**	250 (34)	238 (30)	
**Smoking**			**<.001**
** Current**	57 (8)	57 (7)	
** Former**	484 (66)	445 (57)	
** Never**	196 (27)	285 (36)	
**Long-term health conditions**			
** None**	205 (30)	181 (25)	.088
** 1-2**	402 (59)	456 (63)	
** 3 or more**	72 (11)	87 (12)	
**Educational attainment by age 26**			**<.001**
** None**	185 (26)	196 (26)	
** Up to O-level or equivalent**	145 (21)	283 (37)	
** A-level or equivalent**	228 (33)	222 (29)	
** Degree or higher**	143 (20)	57 (8)	

Abbreviations: BMI, body mass index, NHSD, National Survey of Health and Development; PFS, Pittsburgh Fatigability Scale, SD, standard deviation. Bold typeface indicates statistical significance at the 0.05 level.

a Analyzed with *t* test for continuous variables and chi-square test for categorical variables.

Comparisons of the characteristics of participants included in the analyses with those excluded are shown in Table S1. The participants included had overall higher IC and education levels, were more physically active, and were less likely to be smokers. The men included had a lower mean PFS Mental score compared to those excluded but did not differ by PFS Physical score.

### Networks of physical fatigability, physical activity, and IC

**Figure 1.**
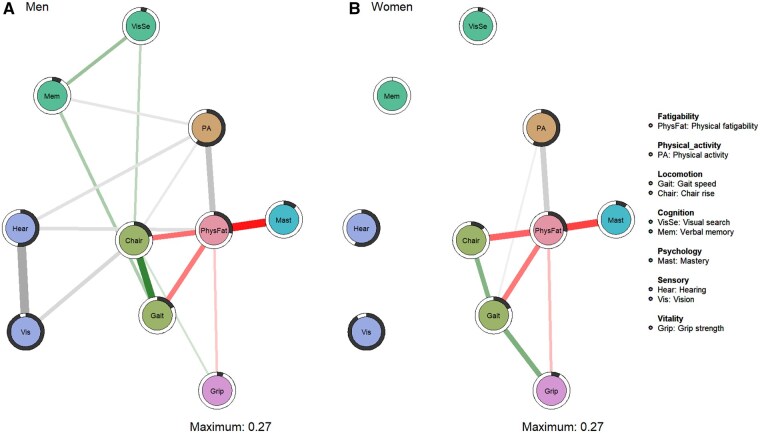
Networks of the relations among physical fatigability, physical activity, and intrinsic capacity (IC) domains for (A) men (*n* = 743) and (B) women (*n* = 794). Green edges indicate positive associations between variables, red edges negative associations, and grey edges associations involving categorical variables. The thickness of an edge reflects the magnitude of the association and black pie charts around the nodes the predictability of the variables.

The networks showing the associations among PFS Physical score, physical activity, and IC domains without covariates are shown in Figure 1. In both sexes, physical fatigability emerged as the most connected node in the network, with 6 other nodes directly connected in men and 5 in women. This node exhibited the highest strength centrality within the network (Figure 3). Higher PFS Physical score was associated with lower mastery, and this was the strongest association for both sexes in the networks [edge weight in men = 0.25 and women = 0.20]. In addition, higher PFS Physical score was associated with less frequent physical activity [men = 0.19 and women = 0.15] as well as slower gait speed [men = 0.14 and women 0.14], chair rise speed [men = 0.16 and women = 0.17], and weaker grip strength [0.05 and 0.07]. Stability analyses indicated robust associations between these nodes (Figures S3 and S4, see online supplementary material for a color version of these figures). The associations also persisted after adjusting for BMI, education, smoking, and long-term health conditions, except for the association between perceived physical fatigability and grip strength in men (Figure S5, see online supplementary material for a color version of this figure). Higher PFS Physical score was also weakly associated with a greater likelihood of hearing difficulties in men [0.10], but the association was less stable. The cognitive domain (verbal memory and visual search) was only indirectly associated with physical fatigability via locomotion indicators in men. In women, cognitive and sensory variables remained disconnected from the other nodes in the network. The directly connected nodes explained 28% of the variance in physical fatigability in men and 26% in women, indicating a moderate but meaningful proportion of variability accounted for by these factors.

The structure of the network shows that the associations of gait speed, chair rise, grip strength, and mastery with physical activity were mainly linked via physical fatigability in both sexes, although chair rise speed in men and gait speed in women maintained a weak direct independent association with physical activity. This network structure persisted after adjusting for BMI, education, smoking, and long-term health conditions (Figure S5, see online supplementary material for a color version of this figure). In addition, mediation models using the SEM framework (Tables S2 and S3) confirmed the mediating role of physical fatigability in these associations, supporting the robustness of the findings.

### Networks of mental fatigability, physical activity, and IC

**Figure 2.**
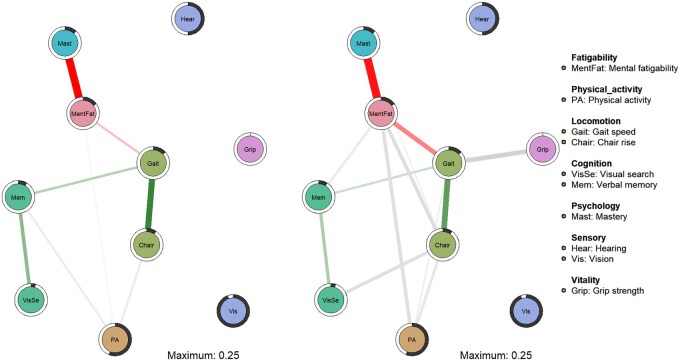
Networks of the relations among mental fatigability, physical activity, and intrinsic capacity (IC) domains for (A) men (*n* = 694) and (B) women (*n* = 746). Green edges indicate positive associations between variables, red edges negative associations, and grey edges associations involving categorical variables. The thickness of an edge reflects the magnitude of the association and black pie charts around the nodes the predictability of the variables.

**Figure 3.**
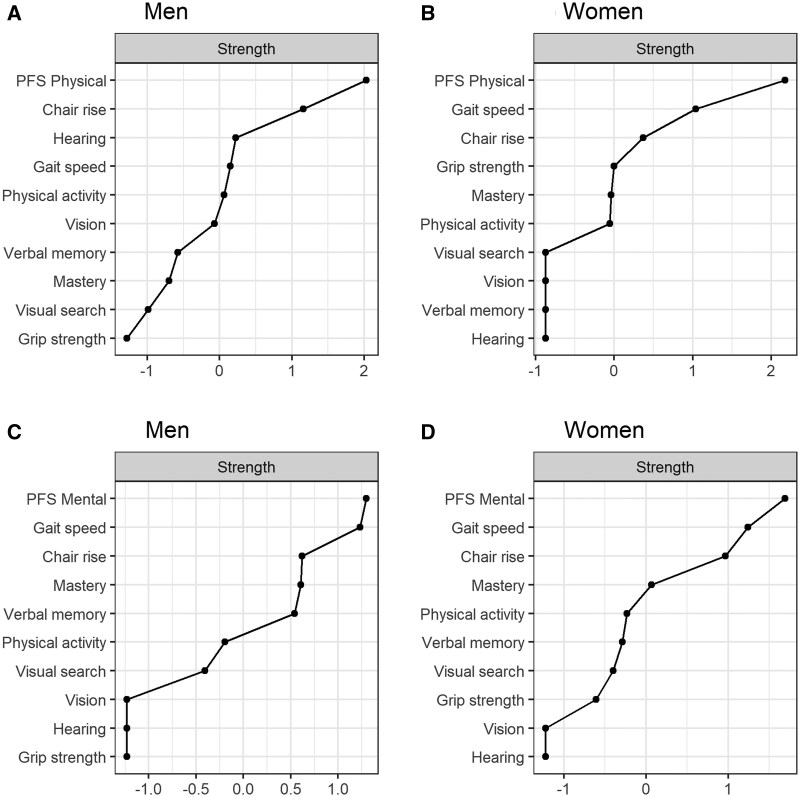
Strength centrality of the nodes in (A) physical fatigability network in men, (B) physical fatigability network in women, (C) mental fatigability network in men, and (D) mental fatigability network in women. PFS, Pittsburgh Fatigability Scale.

Similar to the network including PFS Physical score, PFS Mental score was the most connected node in the network for women and exhibited the highest strength centrality in both sexes (Figures 2 and 3). Higher PFS Mental score had the strongest direct associations with lower mastery in both sexes [edge weight in men = 0.25 and women = 0.23], which also showed good stability in the bootstrap analyses (Figures S6 and S7, see online supplementary material for a color version of these figures) and persisted after covariate adjustment (Figure S8, see online supplementary material for a color version of this figure).

In men, mental fatigability was less connected to other variables compared to physical fatigability. In addition to mastery, in men, PFS Mental score was associated with gait speed [edge weight = 0.06] and physical activity [0.03], but these associations vanished after covariate adjustment. In women, additional associations were found with gait speed [edge weight = 0.12] and chair rise speed [0.10], which showed adequate stability and persisted after covariate adjustment (Figures S6-S8, see online supplementary material for a color version of these figures). Also, we observed a weak but less stable association between mental fatigability and physical activity [0.09] and verbal memory [0.06]. The directly connected nodes in the network unadjusted for covariates explained 15% of the variation in mental fatigability in men and 19% in women, indicating that these factors account for a smaller but still meaningful proportion of variability compared to physical fatigability. Sensory variables and grip strength in men remained disconnected from the other nodes in the network.

The structure of the networks revealed that, in women, mental fatigability served as a mediator in the indirect associations between mastery and physical activity, as well as between locomotion indicators and physical activity. This structure did not change after covariate adjustment (Figure S8, see online supplementary material for a color version of this figure). The indirect associations between mastery and physical activity through mental fatigability were similar to those mediated by physical fatigability, but the associations between locomotion and physical activity were somewhat weaker, as demonstrated in the SEM mediation models (Tables S3 and S4).

Findings for the networks including both, PFS Physical and Mental, were very similar, and the conclusions remained unchanged when the main models were rerun, excluding participants with imputed PFS items (Figures S9 and S10, see online supplementary material for a color version of these figures).

## Discussion

In this study, we have applied a multivariate network approach to explore the complex associations between perceived fatigability, the 5 domains of IC and physical activity in adults aged 68-69 years. Our results revealed that physical and mental fatigability were conditionally associated with 2 of 5 domains of IC (psychology and locomotion) and physical activity, although additional marginal connections with other IC domains were also found. The structure of the physical fatigability network showed that locomotion indicators, mastery, and physical activity behavior were linked to each other through physical fatigability. The same was observed for mental fatigability, but only in women, where the indirect connections were somewhat weaker compared to physical fatigability. These results suggest that perceived physical fatigability may be an important factor on the pathway between reduced physical and mental resources and physical activity behavior in older adults.

The current results align with the findings of a previous cross-sectional study reporting physical fatigability as a likely mediator between lower physical activity levels and slower gait speed in older adults.^5^ Unfortunately, our cross-sectional design does not allow us to establish temporality and so the direction of these associations is unclear. Previous longitudinal analyses have found that physical activity declines may precede changes in perceived physical and mental fatigability levels,^38^^,^^39^ and increasing physical fatigability may precede declines in walking capacity.^7^ However, it is also possible that greater fatigability drives declines in physical activity due to reduced physiological and psychological reserve capacity to preserve energy, but the lack of longitudinal data on the relatively new concept of perceived fatigability has limited research into this in prospective cohort studies. Therefore, although several prospective studies have shown that physical activity precedes fatigability, there is limited understanding of the potentially bidirectional associations between fatigability and physical activity. Recent studies have provided evidence that lower physical fitness, which may also be affected by the normal aging process and burden of diseases, more likely affects reduced physical activity than vice versa.^40^^,^^41^ Nevertheless, instead of focusing on defining determinants and outcomes, the associations are likely multidirectional and further studies with longitudinal data and time-series data from daily life are needed to investigate potential vicious circles or feedback loops between reduced physical and mental reserves, perceived fatigability, and activity behavior affecting the functional aging and disablement, which may not occur in a linear way.^42^^,^^43^

The consistent associations of PFS Physical and Mental scores with mastery also suggest that the experience of fatigability is related not only to reduced physiological but also psychological reserves. This is supported by a previous study reporting associations of higher perceived physical fatigability with higher depressive symptoms.^44^ However, contrary to our expectations, the PFS Mental score was not directly associated with cognitive functioning. This partly contrasts with earlier findings showing associations between PFS Mental and executive function.^10^ Burke et al.^45^ previously found no associations between PFS Mental score and task-based cognitive fatigability suggesting that perceived mental fatigability and fatigability related to mental performance are different constructs. It is possible that perceived mental fatigability reflects factors such as motivation and the effort-reward balance related to a given activity, rather than actual cognitive slowing associated with performance-based cognitive fatigability.^10^

It is also noteworthy that women experienced more fatigability than men, which is consistent with previous research on older age groups.^44^^,^^46^ Beyond differences in prevalence, our network analysis revealed that mental fatigability was more frequently connected to other variables in women than in men, suggesting a broader influence on their functioning and daily activities. This sex difference may reflect distinct biological, psychosocial, and behavioral mechanisms.^47^ Biologically, for example, higher systemic inflammation and greater burden of physical and mental health conditions in older women^48^ may contribute to heightened fatigue perception and energy dysregulation. Psychosocially, factors such as affect regulation and gendered symptom perception could influence symptom reporting and the subjective experience of fatigability. These sex differences are important to consider in future studies.

In this study, we quantified the IC domain of vitality using grip strength, which is often used as an indicator when operationalizing this domain as the measurement is widely available in epidemiological studies. However, there is still a lack of consensus on measuring vitality, which conceptually represents the biological and physiological homeostasis regulation mechanisms affecting all the other, more overt IC domains,^49^ which grip strength alone may not fully capture. Therefore, several other indicators have also been proposed, including fatigability as it reflects an individual’s energy availability.^18^ It was interesting that in our analyses, instead of grip strength, physical fatigability was the most connected node in the networks, indicating strong links with multiple domains of functioning. This suggests that, rather than acting only as a mechanism between reduced reserves and physical activity, fatigability may also reflect underlying aging-related processes in various body functions. For example, a previous study found that higher BMI and greater levels of circulating inflammatory markers were associated with higher perceived physical fatigability,^50^ ­suggesting that fatigability may indicate underlying chronic low-grade inflammation, which is one potential contributor to age-related processes and exhaustion of physiological reserves. Therefore, fatigability could potentially serve as an indicator of vitality, encompassing the often-overlooked mental aspect of vitality. To resolve this conceptual discrepancy, future IC work might differentiate fatigability’s role as a symptom of low physiological reserves from its mechanistic role in mediating age-related changes. Additionally, longitudinal studies incorporating repeated fatigability assessments alongside other IC domains and health outcomes could help disentangle whether fatigability primarily reflects diminished vitality or drives downstream functional outcomes.

The novelty of our study lies in network modelling, which allowed us to efficiently visualize and analyze simultaneous statistical associations within the complex system of IC, fatigability, and physical activity. This approach moves beyond traditional single-variable analyses by revealing conditional dependencies within a complex system, offering new insights into pathways linking physiological and psychological factors with behavior in aging. Rather than focusing on domain-specific outcomes, network modeling allows us to understand the mechanisms of the whole system. Another major strength of our study is the relatively large population-based sample, which was reasonably balanced in terms of the sex of the participants and included a wide range of information on different domains of health and functioning. Further, the measures of physical and mental fatigability have demonstrated good validity and reliability in older adults,^10^^,^^17^ and the PFS overcomes some important limitations of other self-report fatigue measures by including activities that older adults commonly perform and normalizing activities to an intensity level and duration.

The cross-sectional setting is our study’s main limitation, providing only a group-level snapshot of multiple constantly unfolding dynamic processes that occur over different temporal scales, which can be very difficult to untangle even with repeated data. However, the network models applied at one time point can be considered as the first step for identifying which components of the system are central to a problem or phenomenon at hand.^51^ Secondly, although the participation rate in the longitudinal NSHD study has remained high throughout the participants’ lives,^15^ those with lower levels of SEP and poorer health are more likely to have been lost to follow-up,^52^ which may have resulted in selection bias. Thirdly, the MGM models required complete data, which led to the exclusion of individuals with poorer health from the analytical sample (Table S1). This selective attrition and exclusion may have led to an underrepresentation of more vulnerable individuals in the analytical sample, potentially attenuating the observed associations and limiting generalizability to healthier participants. This is an important, though common, limitation in prospective cohort studies. Although assigning the lowest quintile value to nonperformers is a pragmatic approach to reduce missing data and minimize bias, it may have affected variable distributions and either attenuated or inflated associations. A further limitation is the low number of cases in some variable categories, such as physical activity and sensory functions, both of which were based on self-reports. In particular, the physical activity measure may be subject to recall bias and not capture all relevant variability in physical activity, which could affect the accuracy of categorization and the variability of responses. Therefore, the current results should be confirmed in further studies where information on physical activity frequency and intensity has been collected with more detailed and formally validated methods.

To conclude, the present findings suggest that perceived physical fatigability may be an important pathway between reduced physical and mental capacity and physical activity behavior in older age. Future research is warranted to investigate the temporal dynamics between these factors, which could help identify effective targets for interventions to prevent cascades of functional and physical activity decline in later life. However, incorporating fatigability into routine assessments of older adults could help tailor strategies to maintain physical activity and IC, thereby supporting healthy aging.

## Supplementary Material

glaf192_Supplementary_Data

## Data Availability

NSHD data used in this study are available to bona fide researchers upon request to the NSHD Data Sharing Committee via a standard application procedure. Further details can be found at: http://www.nshd.mrc.ac.uk/data; doi: 10.5522/NSHD/Q103

## References

[1] Eldadah BA. Fatigue and fatigability in older adults. PM&R. 2010;2:406-413. 10.1016/j.pmrj.2010.03.022 20656622

[2] Glynn NW , Gmelin T, Renner SW, et al Perceived physical fatigability predicts all-cause mortality in older adults. J Gerontol A Biol Sci Med Sci. 2022;77:837-841. 10.1093/gerona/glab374 34908118 PMC8974332

[3] Kratz AL , Murphy SL, Braley TJ, et al Development of a person-centered conceptual model of perceived fatigability. Qual Life Res. 2019;28:1337-1347. 10.1007/s11136-018-2093-z 30604341 PMC7395299

[4] Gill TM , Desai MM, Gahbauer EA, Holford TR, Williams CS. Restricted activity among community-living older persons: incidence, precipitants, and health care utilization. Ann Intern Med. 2001;135:313. 10.7326/0003-4819-135-5-200109040-00007 11529694

[5] Qiao YS , Gmelin T, Renner SW, et al Evaluation of the bidirectional relations of perceived physical fatigability and physical activity on slower gait speed. J Gerontol A Biol Sci Med Sci. 2021;76:e237-e244. 10.1093/gerona/glaa281 33170216 PMC8436994

[6] Cesari M , Araujo de Carvalho I, Amuthavalli Thiyagarajan J, et al Evidence for the domains supporting the construct of intrinsic capacity. J Gerontol A Biol Sci Med Sci. 2018;73:1653-1660. 10.1093/gerona/gly011 29408961

[7] Simonsick EM , Glynn NW, Jerome GJ, Shardell M, Schrack JA, Ferrucci L. Fatigued, but not frail: perceived fatigability as a marker of impending decline in mobility-intact older adults. J Am Geriatr Soc. 2016;64:1287-1292. 10.1111/jgs.14138 27253228 PMC4914474

[8] Simonsick EM , Schrack JA, Santanasto AJ, Studenski SA, Ferrucci L, Glynn NW. Pittsburgh fatigability scale: one‐page predictor of mobility decline in mobility‐intact older adults. J Am Geriatr Soc. 2018;66:2092-2096. 10.1111/jgs.15531 30315707 PMC6322394

[9] Palmberg L , Rantalainen T, Rantakokko M, et al The associations of activity fragmentation with physical and mental fatigability among community-dwelling 75-, 80-, and 85-year-old people. J Gerontol A Biol Sci Med Sci. 2020;75:e103-e110. 10.1093/gerona/glaa166 32614396

[10] Renner SW , Bear TM, Brown PJ, et al Validation of perceived mental fatigability using the Pittsburgh Fatigability Scale. J Am Geriatr Soc. 2021;69:1343-1348. 10.1111/jgs.17017 33469914 PMC8127403

[11] Koivunen K , Schaap LA, Hoogendijk EO, Schoonmade LJ, Huisman M, van Schoor NM. Exploring the conceptual framework and measurement model of intrinsic capacity defined by the World Health Organization: a scoping review. Ageing Res Rev. 2022;80:101685. 10.1016/j.arr.2022.101685 35830956

[12] Koivunen K , Lindeman K, Välimaa M, Rantanen T. Investigating resilience through intrinsic capacity networks in older adults. J Gerontol A Biol Sci Med Sci. 2024;79:glae048. 10.1093/gerona/glae048 PMC11372706 38366153

[13] Borsboom D , Cramer AOJ. Network analysis: an integrative approach to the structure of psychopathology. Annu Rev Clin Psychol. 2013;9:91-121. 10.1146/annurev-clinpsy-050212-185608 23537483

[14] Kuh D , Pierce M, Adams J, et al Cohort profile: updating the cohort profile for the MRC National Survey of Health and Development: a new clinic-based data collection for ageing research. Int J Epidemiol. 2011;40:e1-e9. 10.1093/ije/dyq231 21345808 PMC3043283

[15] Kuh D , Wong A, Shah I, et al The MRC National Survey of Health and Development reaches age 70: maintaining participation at older ages in a birth cohort study. Eur J Epidemiol. 2016;31:1135-1147. 10.1007/s10654-016-0217-8 27995394 PMC5206260

[16] Wadsworth M , Kuh D, Richards M, Hardy R. Cohort profile: the 1946 National Birth Cohort (MRC National Survey of Health and Development). Int J Epidemiol. 2006;35:49-54. 10.1093/ije/dyi201 16204333

[17] Glynn NW , Santanasto AJ, Simonsick EM, et al The Pittsburgh Fatigability Scale for older adults: development and validation. J Am Geriatr Soc. 2015;63:130-135. 10.1111/jgs.13191 25556993 PMC4971882

[18] Bautmans I , Knoop V, Amuthavalli Thiyagarajan J, et al WHO working definition of vitality capacity for healthy longevity monitoring. Lancet Healthy Longev. 2022;3:e789-e796. 10.1016/S2666-7568(22)00200-8 36356628 PMC9640935

[19] Rantanen T , Guralnik JM, Foley D, et al Midlife hand grip strength as a predictor of old age disability. JAMA. 1999;281:558-560. 10.1001/jama.281.6.558 10022113

[20] Veronese N , Honvo G, Amuthavalli Thiyagarajan J, et al Attributes and definitions of locomotor capacity in older people: a World Health Organisation (WHO) locomotor capacity working group meeting report. Aging Clin Exp Res. 2022;34:481-483. 10.1007/s40520-022-02080-5 35133612 PMC8894172

[21] Cooper AJM , Simmons RK, Kuh D, Brage S, Cooper R, NSHD scientific and data collection team. Physical activity, sedentary time and physical capability in early old age: British Birth Cohort Study. Plos One. 2015;10:e0126465. 10.1371/journal.pone.0126465 25961736 PMC4427100

[22] Cooper R , Strand BH, Hardy R, Patel KV, Kuh D. Physical capability in mid-life and survival over 13 years of follow-up: British birth cohort study. Br Med J. 2014;348:g2219-g2219. 10.1136/bmj.g2219 24787359 PMC4004787

[23] De Looze C , Feeney J, Seeher KM, Amuthavalli Thiyagarajan J, Diaz T, Kenny RA. Assessing cognitive function in longitudinal studies of ageing worldwide: some practical considerations. Age Ageing. 2023;52:iv13-iv25. 10.1093/ageing/afad122 37902512 PMC10615066

[24] Tucker-Drob EM. Global and domain-specific changes in cognition throughout adulthood. Dev Psychol. 2011;47:331-343. 10.1037/a0021361 21244145 PMC5374863

[25] Wu Z , Woods RL, Chong TTJ, et al Cognitive trajectories in community-dwelling older adults and incident dementia, disability and death: a 10-year longitudinal study. Front Med. 2022;9:917254. 10.3389/fmed.2022.917254 PMC9271785 35833102

[26] Beard JR , Officer A, De Carvalho IA, et al The World report on ageing and health: a policy framework for healthy ageing. The Lancet. 2016;387:2145-2154. 10.1016/S0140-6736(15)00516-4 PMC4848186 26520231

[27] Pearlin LI , Schooler C. The structure of coping. J Health Soc Behav. 1978;19:2-21. 10.2307/2136319 649936

[28] Crews JE , Campbell VA. Vision impairment and hearing loss among community-dwelling older Americans: implications for health and functioning. Am J Public Health. 2004;94:823-829. 10.2105/AJPH.94.5.823 15117707 PMC1448344

[29] Dodds R , Kuh D, Aihie Sayer A, Cooper R. Physical activity levels across adult life and grip strength in early old age: updating findings from a British birth cohort. Age Ageing. 2013;42:794-798. 10.1093/ageing/aft124 23981980 PMC3809720

[30] Haslbeck JMB , Waldorp LJ. mgm: estimating time-varying mixed graphical models in high-dimensional data. J Stat Softw. 2020;93:1-46. 10.18637/jss.v093.i08

[31] Burger J , Isvoranu A-M, Lunansky G. Reporting standards for psychological network analyses in cross-sectional data. Psychological methods. 2023;28: 806-824. 10.1037/met0000471. 35404629

[32] Tibshirani R. Regression shrinkage and selection via the Lasso. J R Stat Soc Ser B Methodol. 1996;58:267-288. 10.1111/j.2517-6161.1996.tb02080.x

[33] Epskamp S , Borsboom D, Fried EI. Estimating psychological networks and their accuracy: a tutorial paper. Behav Res Methods. 2018;50:195-212. 10.3758/s13428-017-0862-1 28342071 PMC5809547

[34] Epskamp S , Cramer AOJ, Waldorp LJ, Schmittmann VD, Borsboom D. qgraph: network visualizations of relationships in psychometric data. J Stat Softw. 2012;48:1-18. 10.18637/jss.v048.i04

[35] Fruchterman TMJ , Reingold EM. Graph drawing by force‐directed placement. Softw Pract Exp. 1991;21:1129-1164. 10.1002/spe.4380211102

[36] Haslbeck JMB , Waldorp LJ. How well do network models predict observations? On the importance of predictability in network models. Behav Res Methods. 2018;50:853-861. 10.3758/s13428-017-0910-x 28718088 PMC5880858

[37] Rosseel Y. lavaan : an *R* package for structural equation modeling. J Stat Softw. 2012;48:1-36. 10.18637/jss.v048.i02

[38] Moored KD , Qiao YS, Boudreau RM, et al Prospective associations between physical activity and perceived fatigability in older men: differences by activity type and baseline marital status. J Gerontol A Biol Sci Med Sci. 2022;77:2498-2506. 10.1093/gerona/glac030 35134905 PMC9799181

[39] Qiao YS , Moored KD, Boudreau RM, et al Changes in objectively measured physical activity are associated with perceived physical and mental fatigability in older men. J Gerontol A Biol Sci Med Sci. 2022;77:2507-2516. 10.1093/gerona/glac082 35385877 PMC9799193

[40] Karavirta L , Aittokoski T, Pynnönen K, et al Physical determinants of daily physical activity in older men and women. PLoS One. 2025;20:e0314456. 10.1371/journal.pone.0314456. PMC11790164 39899499

[41] Lindeman K , Koivunen K, Rantalainen T, et al Reciprocal associations between relative or absolute physical activity, walking performance and autonomy in outdoor mobility among older adults: a 4-year follow-up. J Aging Health. 2025;37:606-616. 10.1177/08982643241282918. 39259875 PMC12405652

[42] Kok AAL , Huisman M, Giltay EJ, Lunansky G. Adopting a complex systems approach to functional ageing: bridging the gap between gerontological theory and empirical research. Lancet Healthy Longev. 2025;6:100673. 10.1016/j.lanhl.2024.100673 39884294

[43] Verbrugge LM , Jette AM. The disablement process. Soc Sci Med. 1994;38:1-14. 10.1016/0277-9536(94)90294-1 8146699

[44] LaSorda KR , Gmelin T, Kuipers AL, et al Epidemiology of perceived physical fatigability in older adults: the Long Life Family Study. J Gerontol A Biol Sci Med Sci. 2020;75:e81-e88. 10.1093/gerona/glz288 31828303 PMC7494027

[45] Burke SE , Babu Henry Samuel I, Zhao Q, et al Task-based cognitive fatigability for older adults and validation of mental fatigability subscore of Pittsburgh Fatigability Scale. Front Aging Neurosci. 2018;10:327. 10.3389/fnagi.2018.00327 30405396 PMC6202947

[46] Cohen RW , Meinhardt AJ, Gmelin T, et al Prevalence and severity of perceived mental fatigability in older adults: the Long Life Family Study. J Am Geriatr Soc. 2021;69:1401-1403. 10.1111/jgs.17075 33675035 PMC8142668

[47] Bensing JM , Hulsman RL, Schreurs KMG. Gender differences in fatigue: biopsychosocial factors relating to fatigue in men and women. Med Care. 1999;37:1078-1083. 10.1097/00005650-199910000-00011 10524374

[48] Oksuzyan A , Juel K, Vaupel J, Christensen K. Men: good health and high mortality. Sex differences in health and aging. Aging Clin Exp Res. 2008;20:91-102. 10.1007/BF03324754 18431075 PMC3629373

[49] Beard JR , Jotheeswaran AT, Cesari M, Araujo de Carvalho I. The structure and predictive value of intrinsic capacity in a longitudinal study of ageing. BMJ Open. 2019;9:e026119. 10.1136/bmjopen-2018-026119 PMC6830681 31678933

[50] Cooper R , Popham M, Santanasto AJ, Hardy R, Glynn NW, Kuh D. Are BMI and inflammatory markers independently associated with physical fatigability in old age? Int J Obes. 2019;43:832-841. 10.1038/s41366-018-0087-0 PMC6477893 29795469

[51] Fried EI , Cramer AOJ. Moving forward: challenges and directions for psychopathological network theory and methodology. Perspect Psychol Sci. 2017;12:999-1020. 10.1177/1745691617705892 28873325

[52] Stafford M , Black S, Shah I, et al Using a birth cohort to study ageing: representativeness and response rates in the National Survey of Health and Development. Eur J Ageing. 2013;10:145-157. 10.1007/s10433-013-0258-8 23637643 PMC3637651

