# Supplementary material for: The interplay between perceived fatigability, intrinsic capacity, and physical activity: network analysis in a British birth cohort study
Source: J Gerontol A Biol Sci Med Sci. 2025 Sep 2;80(10):glaf192. doi: 10.1093/gerona/glaf192 (PMC12476581; doi:10.1093/gerona/glaf192)
Supplement: glaf192_Supplementary_Data [file glaf192_supplementary_data.docx]

**Supplementary material**

**Contents**

[Supplementary Table 1. Comparison of the participants included and excluded from the analytical sample. 2](#_Toc199924610)

[Supplementary Figure 1. Spearman correlations between the study variables in men. 4](#_Toc199924611)

[Supplementary Figure 2. Spearman correlations between the study variables in women. 5](#_Toc199924612)

[Supplementary Figure 3. Bootstrapped edge weights in the physical fatigability network without covariates in men. 6](#_Toc199924613)

[Supplementary Figure 4. Bootstrapped edge weights in the physical fatigability network without covariates in women. 7](#_Toc199924614)

[Supplementary Figure 5. Networks of the relationships between physical fatigability, physical activity, and intrinsic capacity domains with covariates 8](#_Toc199924615)

[Supplementary Figure 6. Bootstrapped edge weights in the mental fatigability network without covariates in men. 9](#_Toc199924616)

[Supplementary Figure 7. Bootstrapped edge weights in the mental fatigability network without covariates in women 10](#_Toc199924617)

[Supplementary Figure 8. Networks of the relationships between mental fatigability, physical activity, and intrinsic capacity domains with covariates 11](#_Toc199924618)

[Supplementary Figure 9. Networks of the relationships between physical fatigability, physical activity, and intrinsic capacity domains, excluding participants with imputed PFS scores 12](#_Toc199924619)

[Supplementary Figure 10. Networks of the relationships between mental fatigability, physical activity, and intrinsic capacity domains, excluding participants with imputed PFS scores 13](#_Toc199924620)

[Supplementary Table 2. Mediation models in men. 14](#_Toc199924621)

[Supplementary Table 3. Mediation models in women 15](#_Toc199924622)

# Supplementary Table 1. Comparison of the participants included and excluded from the analytical sample.

|  | **Men** | |  | **Women** | |  |
| --- | --- | --- | --- | --- | --- | --- |
|  | **Included** (n=743) | **Excluded** (n=308) | **p-value** | **Included** (n=794) | **Excluded** (n=303) | **p-value** |
|  | **Mean** ± **SD** | **Mean** ± **SD** |  | **Mean** ± **SD** | **Mean** ± **SD** |  |
| PFS Physical score, 0-50 | 12.6 ± 8.4 | 14.4 ± 11.3 | .078 | 15.9 ± 9.2 | 16.8 ± 10.0 | .335 |
| Gait speed (m/s) | 1.1 ± 0.3 | 1.0 ± 0.3 | **<.001** | 1.1 ± 0.3 | 1.0 ± 0.3 | **<.001** |
| Chair rise speed (stand-ups per minute) | 27.8 ± 8.6 | 25.2 ± 8.8 | **<.001** | 26.3 ± 8.1 | 24.7 ± 9.3 | **<.001** |
| Verbal memory | 24.7 ± 5.9 | 19.7 ± 6.0 | **<.001** | 23.8 ± 5.8 | 21.3 ± 6.1 | **<.001** |
| Visual search | 265.5 ± 70.1 | 262.2 ± 80.7 | .545 | 273.5 ± 68.8 | 280.7 ± 78.9 | .147 |
| Mastery | 22.8 ± 3.3 | 21.8 ± 4.3 | **.002** | 21.6 ± 3.6 | 21.1 ± 3.3 | .113 |
| Grip strength (kg) | 40.7 ± 8.5 | 38.5 ± 8.3 | **<.001** | 24.6 ± 5.7 | 22.1 ± 6.0 | **<.001** |
| BMI (kg/m^2^) | 27.9 ± 4.2 | 29.0 ± 5.2 | **.001** | 27.9 ± 5.5 | 28.9 ± 6.3 | **<.001** |
|  | **n (%)** | **n (%)** |  | **n (%)** | **n (%)** |  |
| Near vision |  |  | .137 |  |  | .941 |
| No difficulty | 702 (95) | 281 (92) |  | 721 (91) | 269 (90) |  |
| A little difficulty | 32 (4) | 14 (5) |  | 42 (5) | 16 (5) |  |
| Some or a great deal of difficulty | 9 (1) | 9 (3) |  | 31 (4) | 13 (4) |  |
| Hearing in conversation |  |  | .757 |  |  | .175 |
| No difficulty | 374 (50) | 160 (53) |  | 446 (56) | 183 (61) |  |
| A little difficulty | 212 (29) | 85 (28) |  | 216 (27) | 65 (22) |  |
| Some or a great deal of difficulty | 157 (21) | 59 (19) |  | 132 (17) | 50 (17) |  |
| Physical activity |  |  | **<.001** |  |  | **<.001** |
| None | 394 (53) | 202 (68) |  | 427 (54) | 208 (71) |  |
| 1-4 times/month | 99 (13) | 33 (11) |  | 129 (16) | 36 (12) |  |
| 5 or more times/month | 250 (34) | 62 (21) |  | 238 (30) | 50 (17) |  |
| Smoking |  |  | **.030** |  |  | .148 |
| Current | 57 (8) | 39 (13) |  | 57 (7) | 31 (11) |  |
| Former | 484 (66) | 185 (62) |  | 445 (57) | 169 (57) |  |
| Never | 196 (27) | 76 (25) |  | 285 (36) | 98 (32) |  |
| Long-term health conditions |  |  | .733 |  |  | .061 |
| None | 205 (30) | 73 (28) |  | 181 (25) | 76 (29) |  |
| 1-2 | 402 (59) | 157 (60) |  | 456 (63) | 146 (55) |  |
| 3 or more | 72 (11) | 31 (12) |  | 87 (12) | 43 (16) |  |
| Educational attainment by age 26 |  |  | **<.001** |  |  | **<.001** |
| None | 185 (26) | 133 (45) |  | 196 (26) | 120 (43) |  |
| Up to O-level or equivalent | 145 (21) | 59 (20) |  | 283 (37) | 91 (33) |  |
| A-level or equivalent | 228 (33) | 80 (27) |  | 222 (29) | 68 (22) |  |
| Degree or higher | 143 (20) | 22 (8) |  | 57 (8) | 9 (3) |  |

*Note.* SD = standard deviation, PFS = Pittsburgh Fatigability Scale, BMI = Body Mass Index. * Analysed with t-test for continuous variables and chi-square test for categorical variables.

**
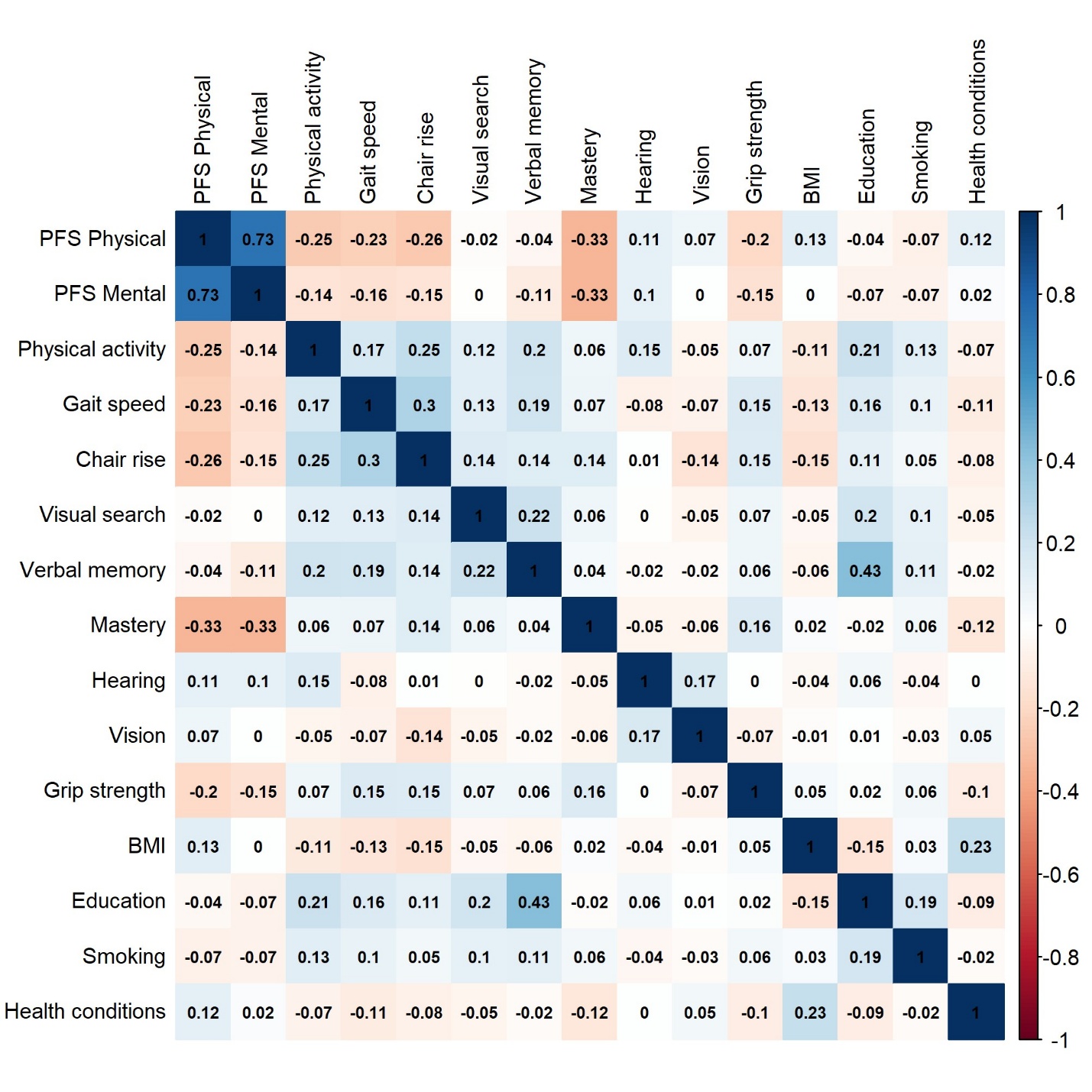
**

# Supplementary Figure 1. Spearman correlations between the study variables in men.


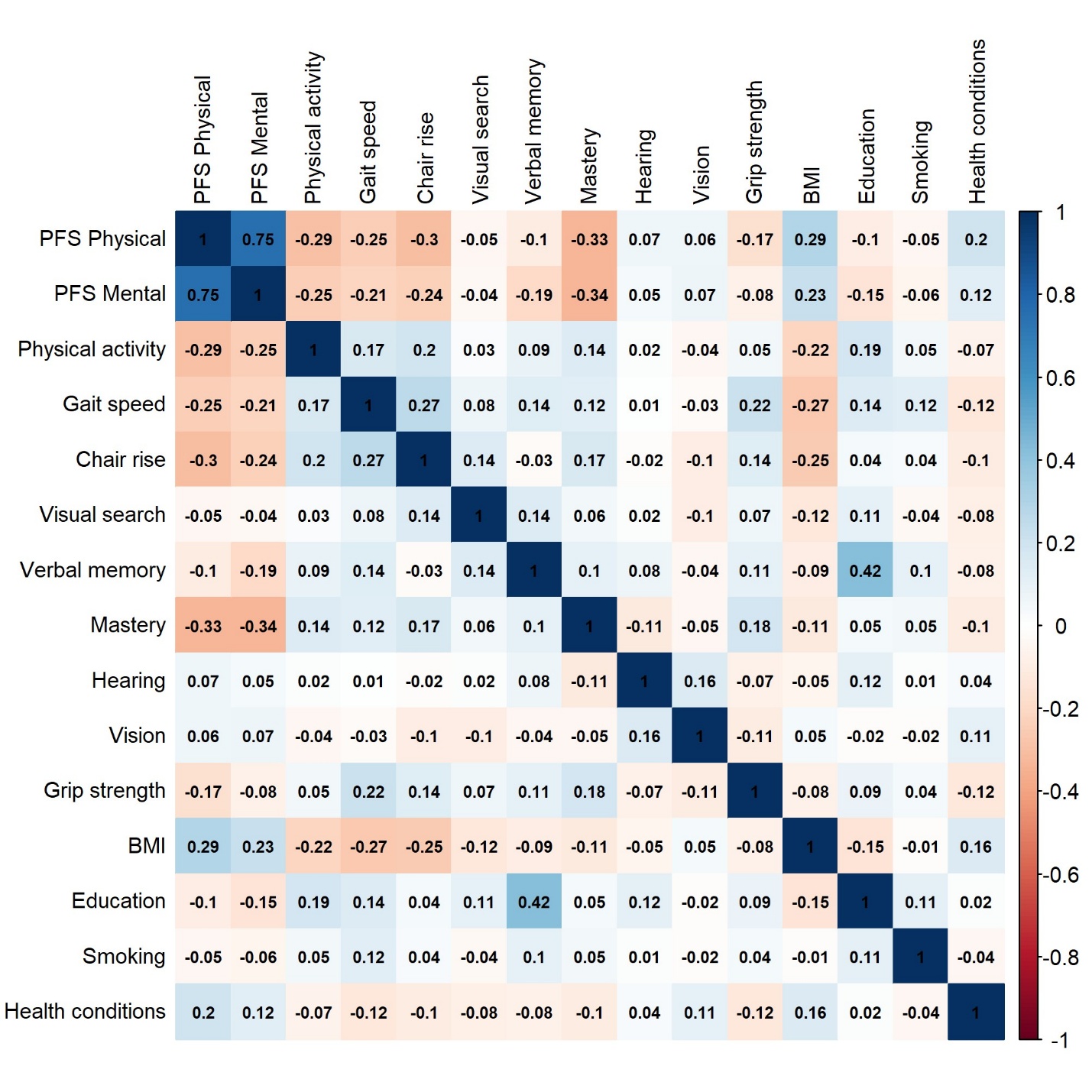


# Supplementary Figure 2. Spearman correlations between the study variables in women.


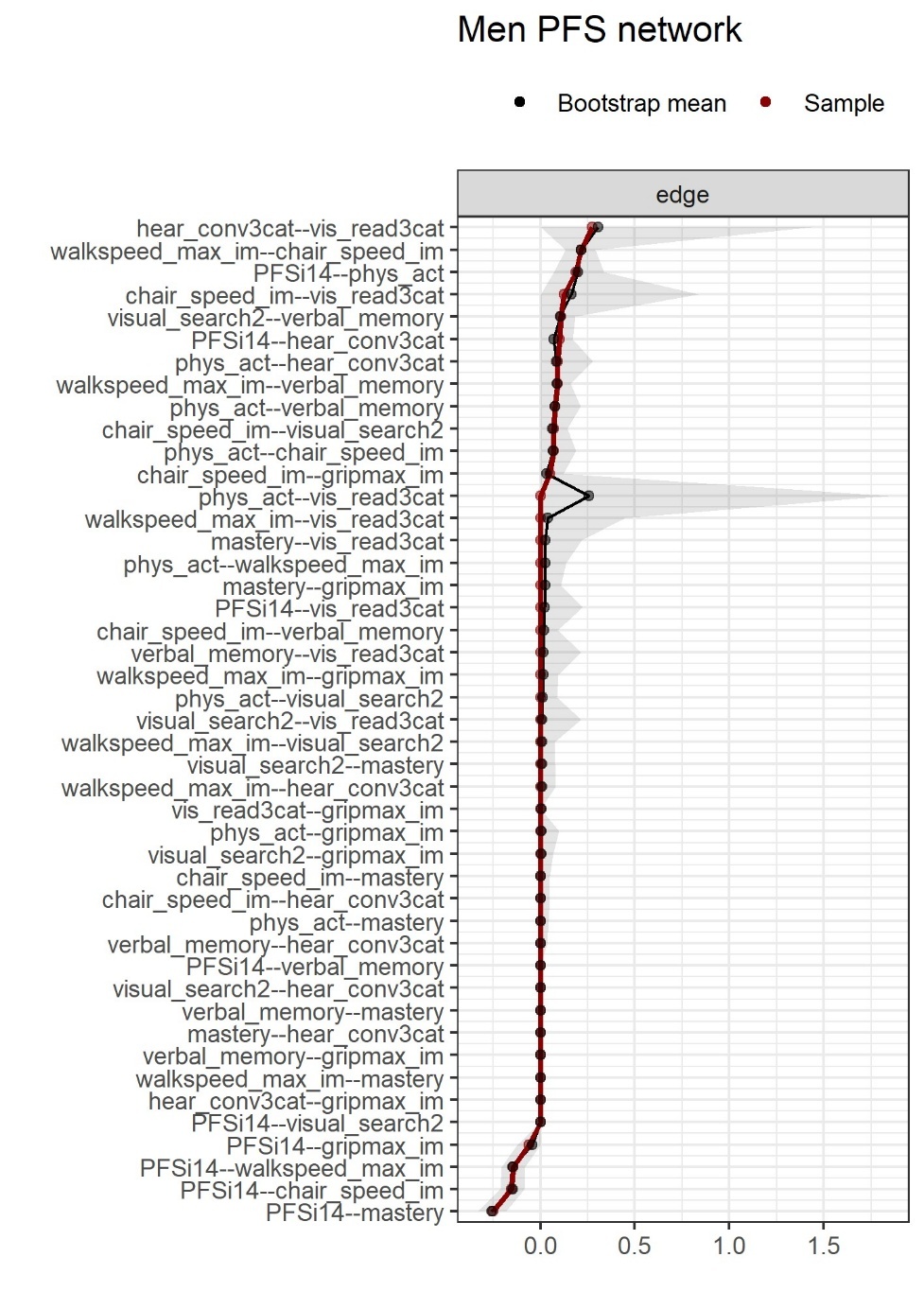


Supplementary Figure 3. Bootstrapped edge weights in the physical fatigability network without covariates in men. PFS = Physical Fatigability Scale.


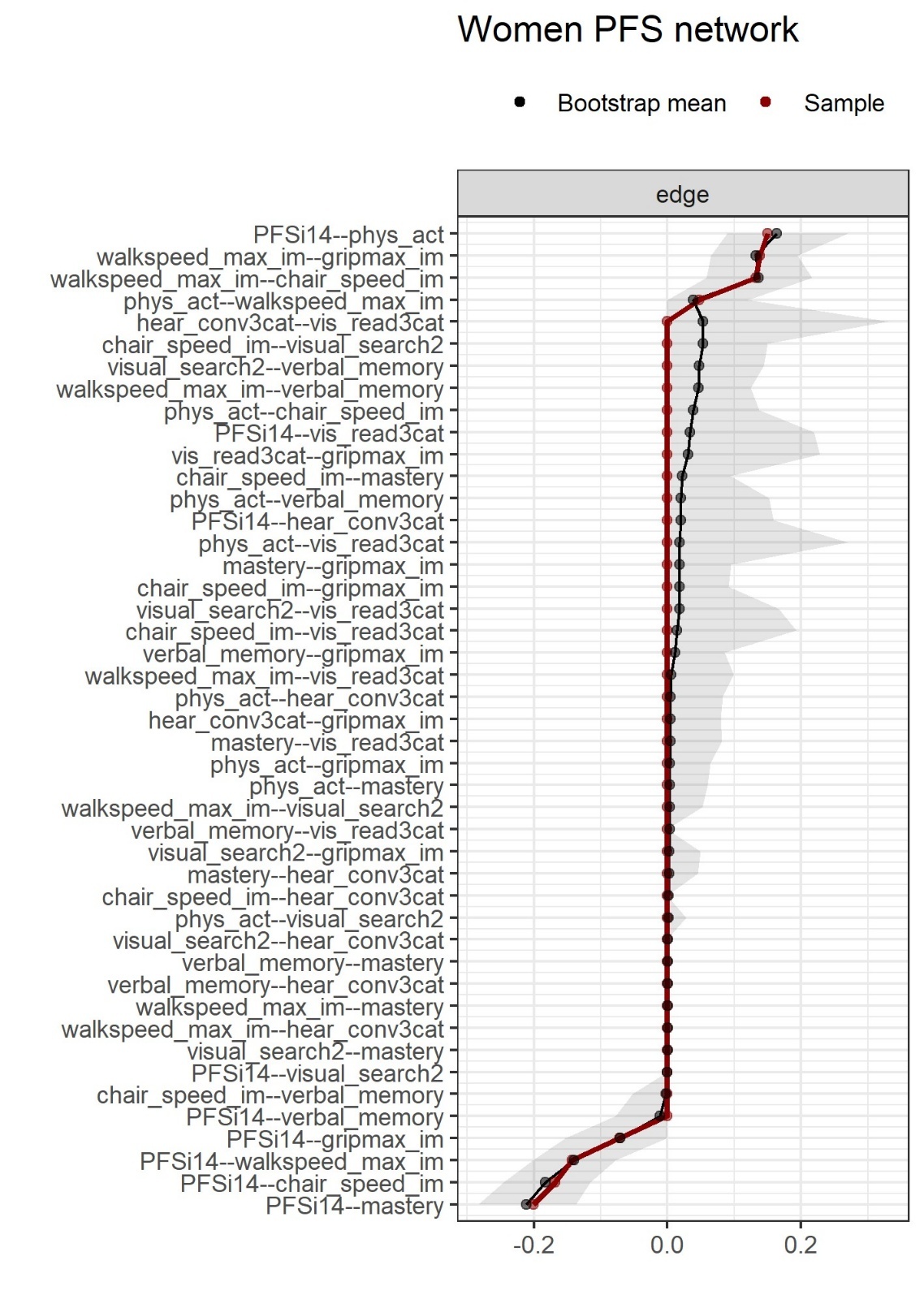


Supplementary Figure 4. Bootstrapped edge weights in the physical fatigability network without covariates in women. PFS = Physical Fatigability Scale.


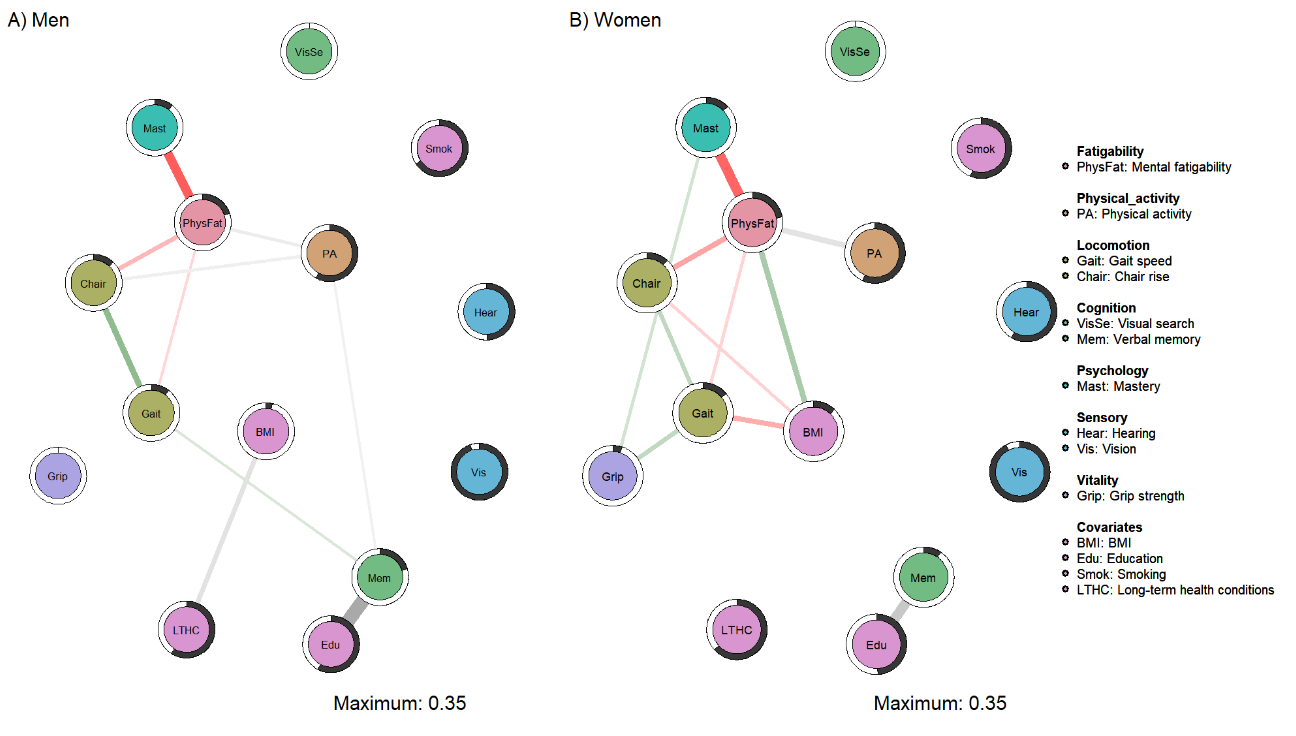


Supplementary Figure 5. Networks of the relationships between physical fatigability, physical activity, and intrinsic capacity domains with covariates for A) Men and B) Women. Green edges indicate positive associations between variables, red edges negative associations, and grey edges associations involving categorical variables. The thickness of an edge reflects the magnitude of the association and black pie charts around the nodes the predictability of the variables.


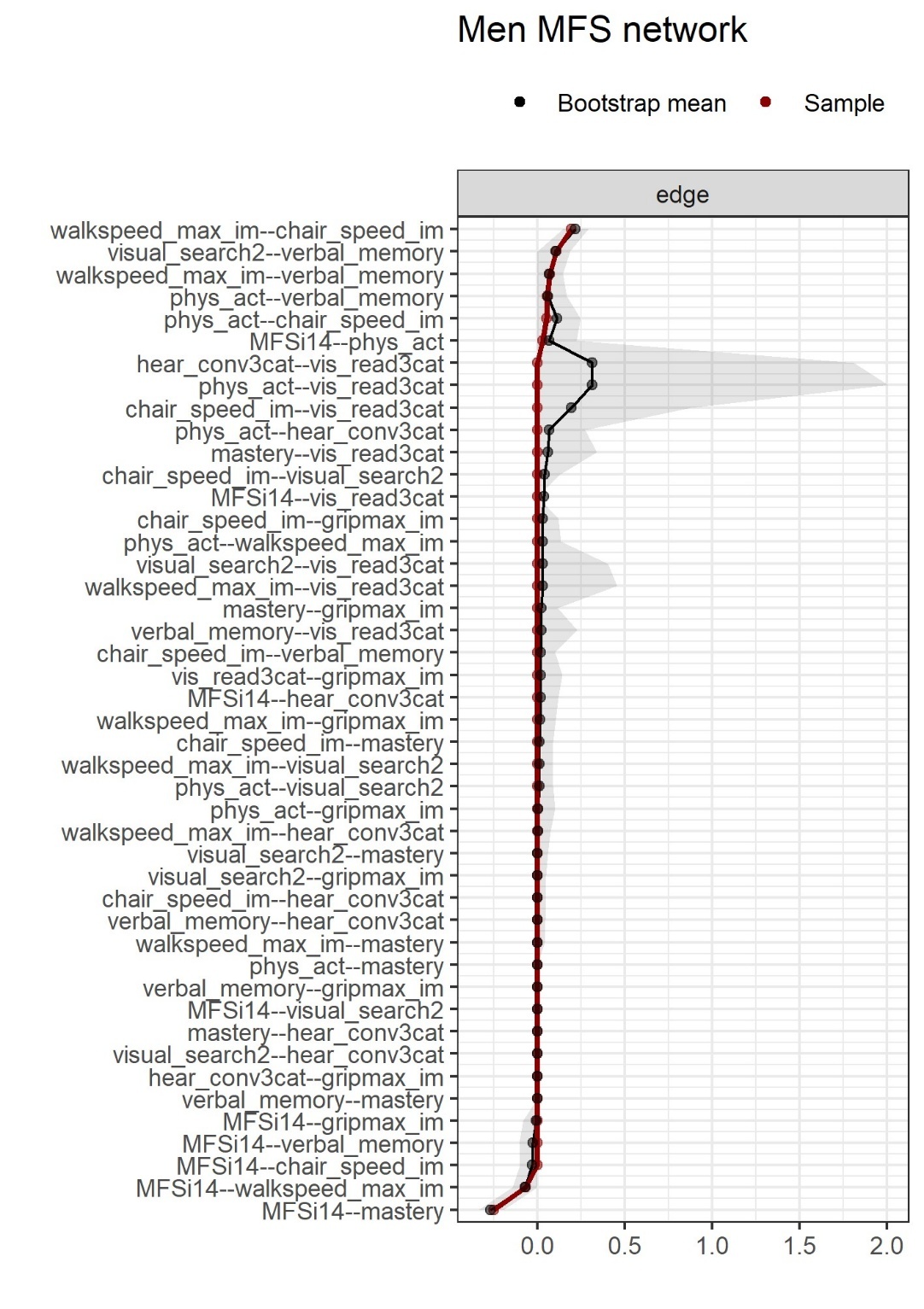


Supplementary Figure 6. Bootstrapped edge weights in the mental fatigability network without covariates in men. MFS = Mental Fatigability Scale.


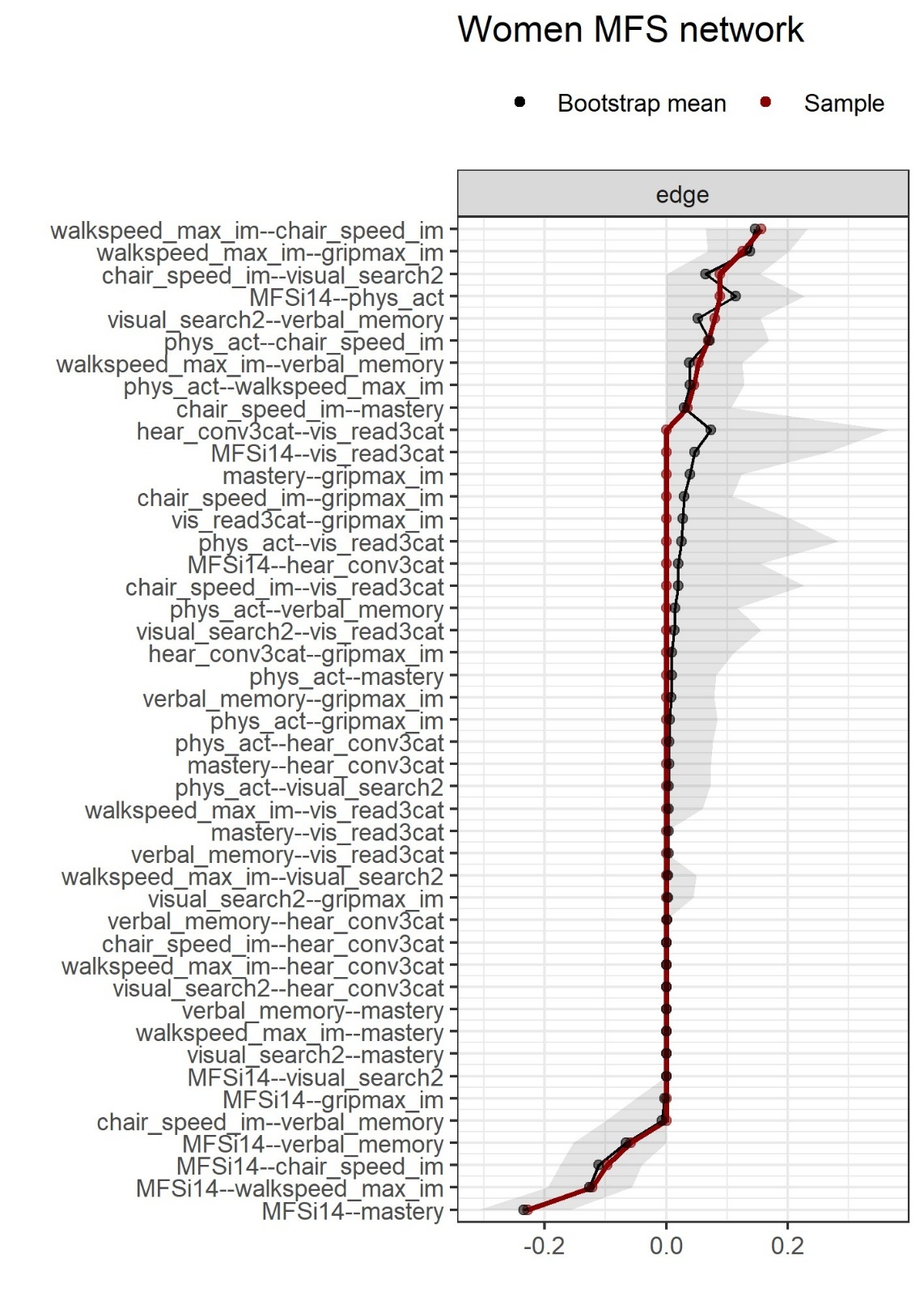


Supplementary Figure 7. Bootstrapped edge weights in the mental fatigability network without covariates in women. MFS = Mental Fatigability Scale.


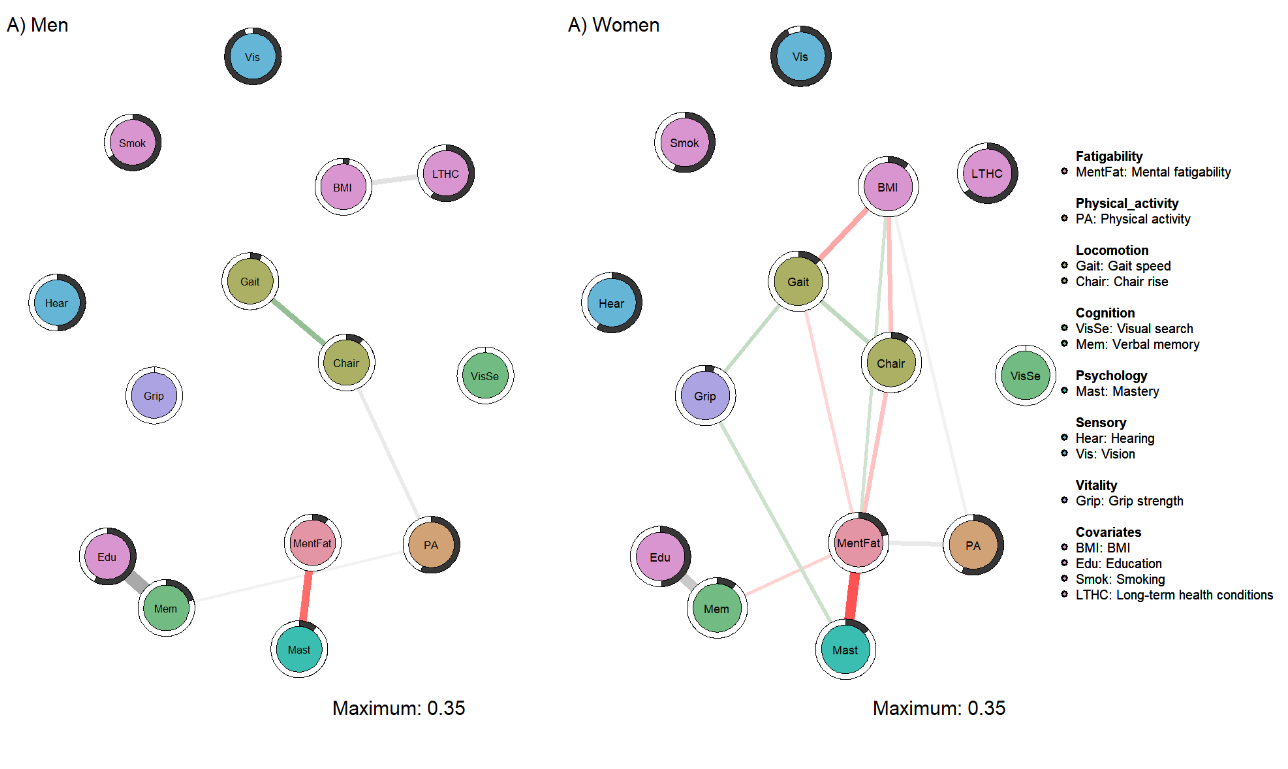


Supplementary Figure 8. Networks of the relationships between mental fatigability, physical activity, and intrinsic capacity domains with covariates for A) Men and B) Women. Green edges indicate positive associations between variables, red edges negative associations, and grey edges associations involving categorical variables. The thickness of an edge reflects the magnitude of the association and black pie charts around the nodes the predictability of the variables.


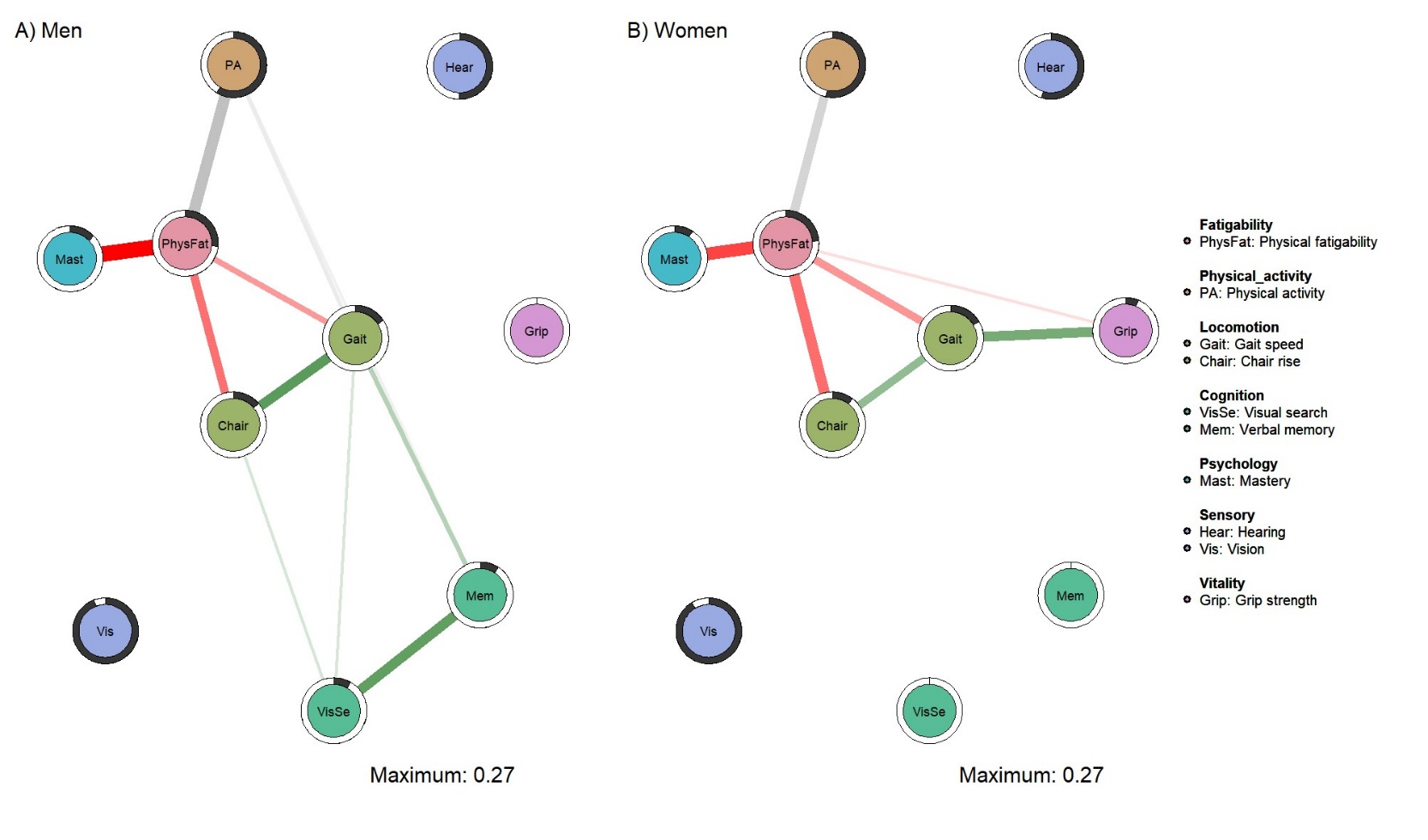


Supplementary Figure 9. Networks of the relationships between physical fatigability, physical activity, and intrinsic capacity domains, excluding participants with imputed PFS scores. A) Men and B) Women. Green edges indicate positive associations between variables, red edges negative associations, and grey edges associations involving categorical variables. The thickness of an edge reflects the magnitude of the association and black pie charts around the nodes the predictability of the variables.


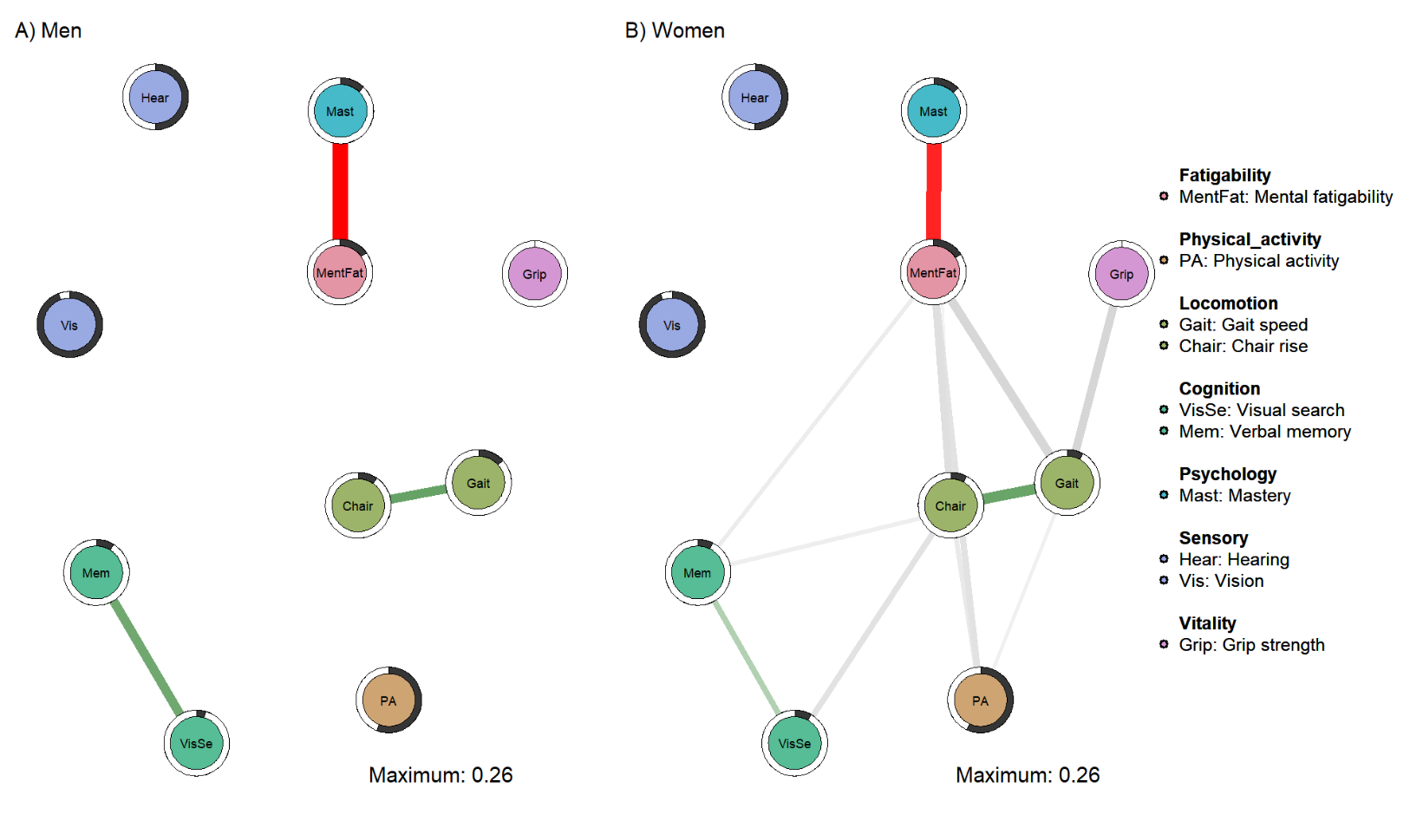


Supplementary Figure 10. Networks of the relationships between mental fatigability, physical activity, and intrinsic capacity domains, excluding participants with imputed PFS scores. A) Men and B) Women. Green edges indicate positive associations between variables, red edges negative associations, and grey edges associations involving categorical variables. The thickness of an edge reflects the magnitude of the association and black pie charts around the nodes the predictability of the variables.

# Supplementary Table 2. Mediation models in men.

|  | **Path X 🡪 M 🡪 Y** | **Path *a*** | **Path *b*** | **Path c´** | **Indirect effect** |
| --- | --- | --- | --- | --- | --- |
|  | **Physical fatigability** | **B (SE)** | **B (SE)** | **B (SE)** | **B (SE)** |
| M1 | Gait speed 🡪 PFS 🡪 PA | -8.136 (0.977)*** | -0.035 (0.005)*** | 0.401 (0.145)** | 0.286 (0.055)*** |
| M2 | Gait speed 🡪 PFS 🡪 PA | -4.954 (1.019)*** | -0.039 (0.006)*** | 0.161 (0.159) | 0.194 (0.049)*** |
| M1 | Chair rise 🡪 PFS 🡪 PA | -0.312 (0.035)*** | -0.032 (0.005)*** | 0.025 (0.006)*** | 0.010 (0.002)*** |
| M2 | Chair rise 🡪 PFS 🡪 PA | -0.200 (0.038)*** | -0.039 (0.006)*** | 0.019 (0.006)** | 0.008 (0.002)*** |
| M1 | Grip strength 🡪 PFS 🡪 PA | -0.184 (0.032)*** | -0.039 (0.005)*** | 0.000 (0.005) | 0.007 (0.002)*** |
| M2 | Grip strength 🡪 PFS 🡪 PA | -0.081 (0.029) ** | -0.137 (0.004)*** | -0.012 (0.006)* | 0.011 (0.004)* |
| M1 | Mastery 🡪 PFS 🡪 PA | -0.905 (0.080)*** | -0.040 (0.005)*** | -0.014 (0.014) | 0.037 (0.006)*** |
| M2 | Mastery 🡪 PFS 🡪 PA | -0.732 (0.074)*** | -0.039 (0.006)*** | -0.022 (0.014) | 0.029 (0.005)*** |
|  | **Mental fatigability** |  |  |  |  |
| M1 | Gait speed 🡪 MFS 🡪 PA | -.4.945 (0.870)*** | -0.029 (0.007)*** | 0.508 (0.148)** | 0.143 (0.042)** |
| M2 | Gait speed 🡪 MFS 🡪 PA | -2.826 (0.959)** | -0.028 (0.007)*** | 0.246 (0.160) | 0.080 (0.034)* |
| M1 | Chair rise 🡪 MFS 🡪 PA | -0.164 (0.034)*** | -0.027 (0.007)*** | 0.032 (0.006)*** | 0.004 (0.001)** |
| M2 | Chair rise 🡪 MFS 🡪 PA | -0.077 (0.038)* | -0.028 (0.007)*** | 0.026 (0.007)*** | 0.002 (0.001) |
| M1 | Grip strength 🡪 MFS 🡪 PA | -0.104 (0.025)*** | -0.032 (0.007)*** | 0.007 (0.005) | 0.003 (0.001)** |
| M2 | Grip strength 🡪 MFS 🡪 PA | -0.035 (0.027) | -0.028 (0.007)*** | 0.001 (0.006) | 0.001 (0.001) |
| M1 | Mastery 🡪 MFS 🡪 PA | -0.790 (0.073)*** | -0.033 (0.007)*** | -0.005 (0.014) | 0.026 (0.006)*** |
| M2 | Mastery 🡪 MFS 🡪 PA | -0.710 (0.073)*** | -0.028 (0.007)*** | -0.015 (0.015) | 0.020 (0.006)*** |

M1 = Model 1 unadjusted, M2 = adjusted for other intrinsic capacity variables in the network, PFS = Physical fatigability scale, MFS = Mental Fatigability Scale, Path *a* = X 🡪 M, Path *b* = M 🡪 Y, Path c´= X – Y after controlling for the mediator, Indirect effect = *a* *x b*, B = standardized beta, SE = standard error, * < 0.05, **<0.01, *** <0.001

Supplementary Table 3. Mediation models in women.

|  | **Path X 🡪 M 🡪 Y** | **Path *a*** | **Path *b*** | **Path c´** | **Indirect effect** |
| --- | --- | --- | --- | --- | --- |
|  | **Physical fatigability** | **B (SE)** | **B (SE)** | **B (SE)** | **B (SE)** |
| M1 | Gait speed 🡪 PFS 🡪 PA | -10.311 (0.994)*** | -0.036 (0.004)*** | 0.480 (0.147)** | 0.370 (0.056)*** |
| M2 | Gait speed 🡪 PFS 🡪 PA | -5.828 (1.041)*** | -0.125 (0.003)*** | -0.168 (0.179) | 0.729 (0.132)*** |
| M1 | Chair rise 🡪 PFS 🡪 PA | -0.390 (0.035)*** | -0.035 (0.004)*** | 0.017 (0.005)** | 0.014 (0.002)*** |
| M2 | Chair rise 🡪 PFS 🡪 PA | -0.254 (0.036)*** | -0.125 (0.003)*** | -0.008 (0.006) | 0.032 (0.005)*** |
| M1 | Grip strength 🡪 PFS 🡪 PA | -0.387 (0.053)*** | -0.039 (0.004)*** | 0.004 (0.008) | 0.015 (0.003)*** |
| M2 | Grip strength 🡪 PFS 🡪 PA | -0.152 (0.049)** | -0.125 (0.003)*** | -0.018 (0.009)* | 0.019 (0.006)** |
| M1 | Mastery 🡪 PFS 🡪 PA | -0.873 (0.086)*** | -0.038 (0.004)*** | 0.013 (0.012) | 0.033 (0.005)*** |
| M2 | Mastery 🡪 PFS 🡪 PA | -0.633 (0.080)*** | -0.125 (0.003)*** | -0.049 (0.015)** | 0.079 (0.010)*** |
|  | **Mental fatigability** |  |  |  |  |
| M1 | Gait speed 🡪 MFS 🡪 PA | -7.809 (0.961)*** | -0.034 (0.005)*** | 0.533 (0.152)*** | 0.268 (0.053)*** |
| M2 | Gait speed 🡪 MFS 🡪 PA | -4.856 (1.009)*** | -0.028 (0.006)*** | 0.394 (0.162)* | 0.134 (0.040)** |
| M1 | Chair rise 🡪 MFS 🡪 PA | -0.260 (0.035)*** | -0.033 (0.005)*** | 0.023 (0.005)*** | 0.009 (0.002)*** |
| M2 | Chair rise 🡪 MFS 🡪 PA | -0.160 (0.037)*** | -0.139 (0.003)*** | 0.003 (0.007) | 0.022 (0.005)*** |
| M1 | Grip strength 🡪 MFS 🡪 PA | -0.208 (0.049)*** | -0.037 (0.005)*** | 0.010 (0.008) | 0.008 (0.002)*** |
| M2 | Grip strength 🡪 MFS 🡪 PA | -0.007 (0.047) | -0.139 (0.003)*** | 0.000 (0.010) | 0.001 (0.007) |
| M1 | Mastery 🡪 MFS 🡪 PA | -0.789 (0.076)*** | -0.035 (0.005)*** | 0.020 (0.013) | 0.028 (0.005)*** |
| M2 | Mastery 🡪 MFS 🡪 PA | -0.632 (0.074)*** | -0.139 (0.003)*** | -0.055 (0.016)*** | 0.088 (0.010)*** |

M1 = Model 1 unadjusted, M2 = adjusted for other intrinsic capacity variables in the network, PFS = Physical fatigability scale, MFS = Mental Fatigability Scale, Path *a* = X 🡪 M, Path *b* = M 🡪 Y, Path c´= X – Y after controlling for the mediator, Indirect effect = *a* *x b*, B = standardized beta, SE = standard error, * < 0.05, **<0.01, *** <0.001
